# Inflated expectations: Rare-variant association analysis using public controls

**DOI:** 10.1371/journal.pone.0280951

**Published:** 2023-01-25

**Authors:** Jung Kim, Danielle M. Karyadi, Stephen W. Hartley, Bin Zhu, Mingyi Wang, Dongjing Wu, Lei Song, Gregory T. Armstrong, Smita Bhatia, Leslie L. Robison, Yutaka Yasui, Brian Carter, Joshua N. Sampson, Neal D. Freedman, Alisa M. Goldstein, Lisa Mirabello, Stephen J. Chanock, Lindsay M. Morton, Sharon A. Savage, Douglas R. Stewart

**Affiliations:** 1 Division of Cancer Epidemiology and Genetics, National Cancer Institute, Rockville, Maryland, United States of America; 2 Cancer Genomics Research Laboratory, Division of Cancer Epidemiology and Genetics, National Cancer Institute, Rockville, Maryland, United States of America; 3 Leidos Biomedical Research, Inc., Frederick National Laboratory for Cancer Research, Frederick, Maryland, United States of America; 4 Department of Epidemiology and Cancer Control, St. Jude Children’s Research Hospital, Memphis, Tennessee, United States of America; 5 Institute for Cancer Outcomes and Survivorship, University of Alabama at Birmingham, Birmingham, Alabama, United States of America; 6 Department of Population Science, American Cancer Society, Atlanta, Georgia, United States of America; CNR, ITALY

## Abstract

The use of publicly available sequencing datasets as controls (hereafter, “public controls”) in studies of rare variant disease associations has great promise but can increase the risk of false-positive discovery. The specific factors that could contribute to inflated distribution of test statistics have not been systematically examined. Here, we leveraged both public controls, gnomAD v2.1 and several datasets sequenced in our laboratory to systematically investigate factors that could contribute to the false-positive discovery, as measured by λ_Δ95_, a measure to quantify the degree of inflation in statistical significance. Analyses of datasets in this investigation found that 1) the significantly inflated distribution of test statistics decreased substantially when the same variant caller and filtering pipelines were employed, 2) differences in library prep kits and sequencers did not affect the false-positive discovery rate and, 3) joint *vs*. separate variant-calling of cases and controls did not contribute to the inflation of test statistics. Currently available methods do not adequately adjust for the high false-positive discovery. These results, especially if replicated, emphasize the risks of using public controls for rare-variant association tests in which individual-level data and the computational pipeline are not readily accessible, which prevents the use of the same variant-calling and filtering pipelines on both cases and controls. A plausible solution exists with the emergence of cloud-based computing, which can make it possible to bring containerized analytical pipelines to the data (rather than the data to the pipeline) and could avert or minimize these issues. It is suggested that future reports account for this issue and provide this as a limitation in reporting new findings based on studies that cannot practically analyze all data on a single pipeline.

## Introduction

Large-scale, publicly available germline exome and genome sequencing datasets have emerged as invaluable tools for investigating associations between genetic variants and disease. These datasets are frequently used as controls to substantially increase the statistical power for investigation of rare genetic variants that could contribute to specific diseases. Although the method of variant-calling in each resource is generally described (*e*.*g*., Exome Variant Server [1], 1000 Genomes [2], The Exome Aggregation Consortium/The Genome Aggregation Database [gnomAD] [3]), the raw data files and/or pipeline methods typically are not readily accessible. Previous studies have reported that using public controls in rare-variant association analyses can lead to a marked increase in false-positive findings [4, 5]. Although methods have been developed to adjust for this inflation (*e*.*g*., TRAPD [6], ProxECAT [7], iECAT [8]), the performance of these methods in larger datasets and the specific factors that contribute to the inflated distribution of test statistics have not been systematically examined.

## Results

### Overview of λ_Δ95_, analytic approach and sample sets

**Fig 1:**
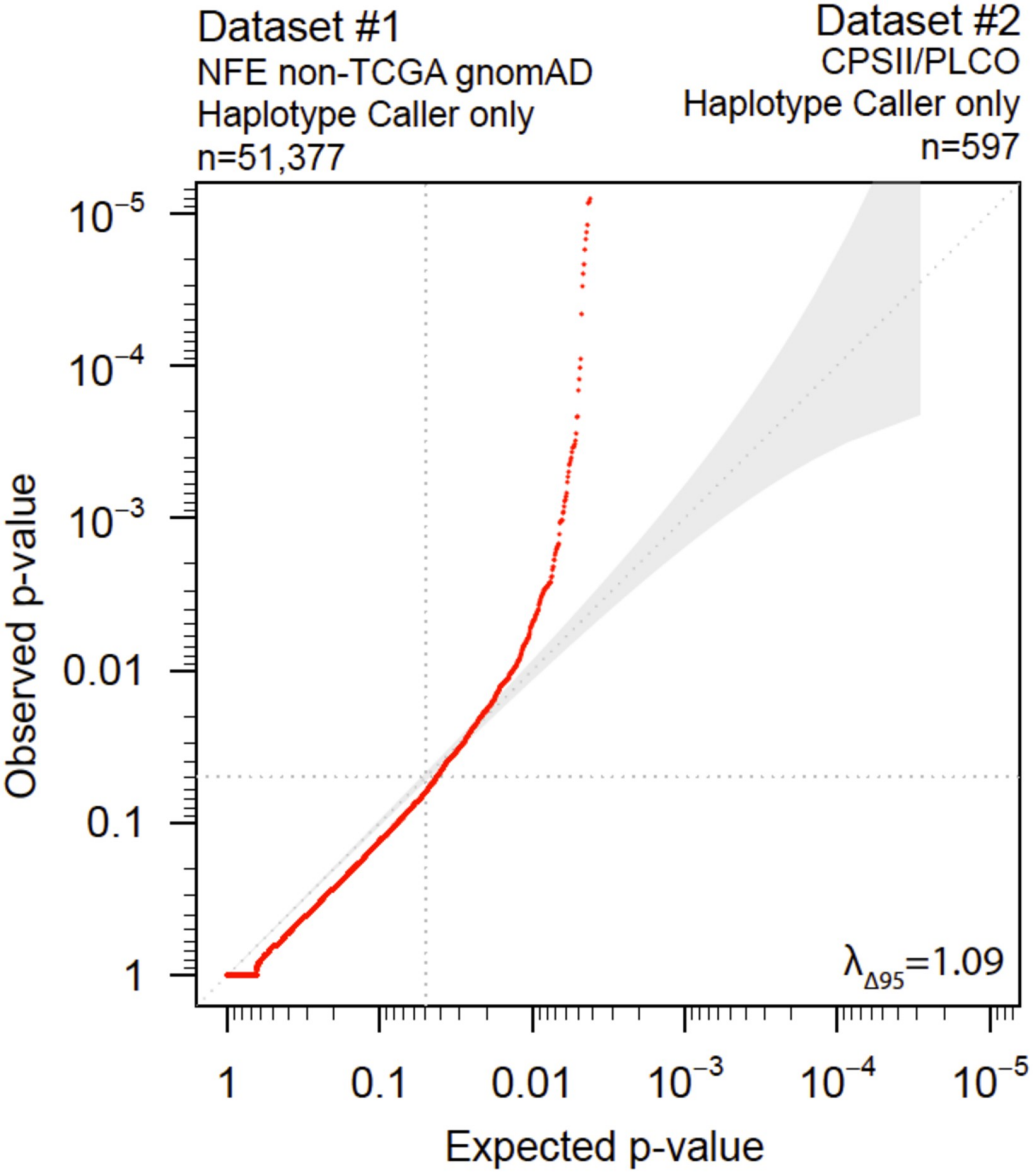
Demonstration of increased false-positive findings with expected-null findings using public controls. Quantile-quantile plot (synonymous variants only) of non-Finnish European non-TCGA (The Cancer Genome Atlas) gnomAD (serving as a public control) versus an experimental dataset. We observed highly inflated p-values deviating from the 95% confidence interval.

**Fig 2:**
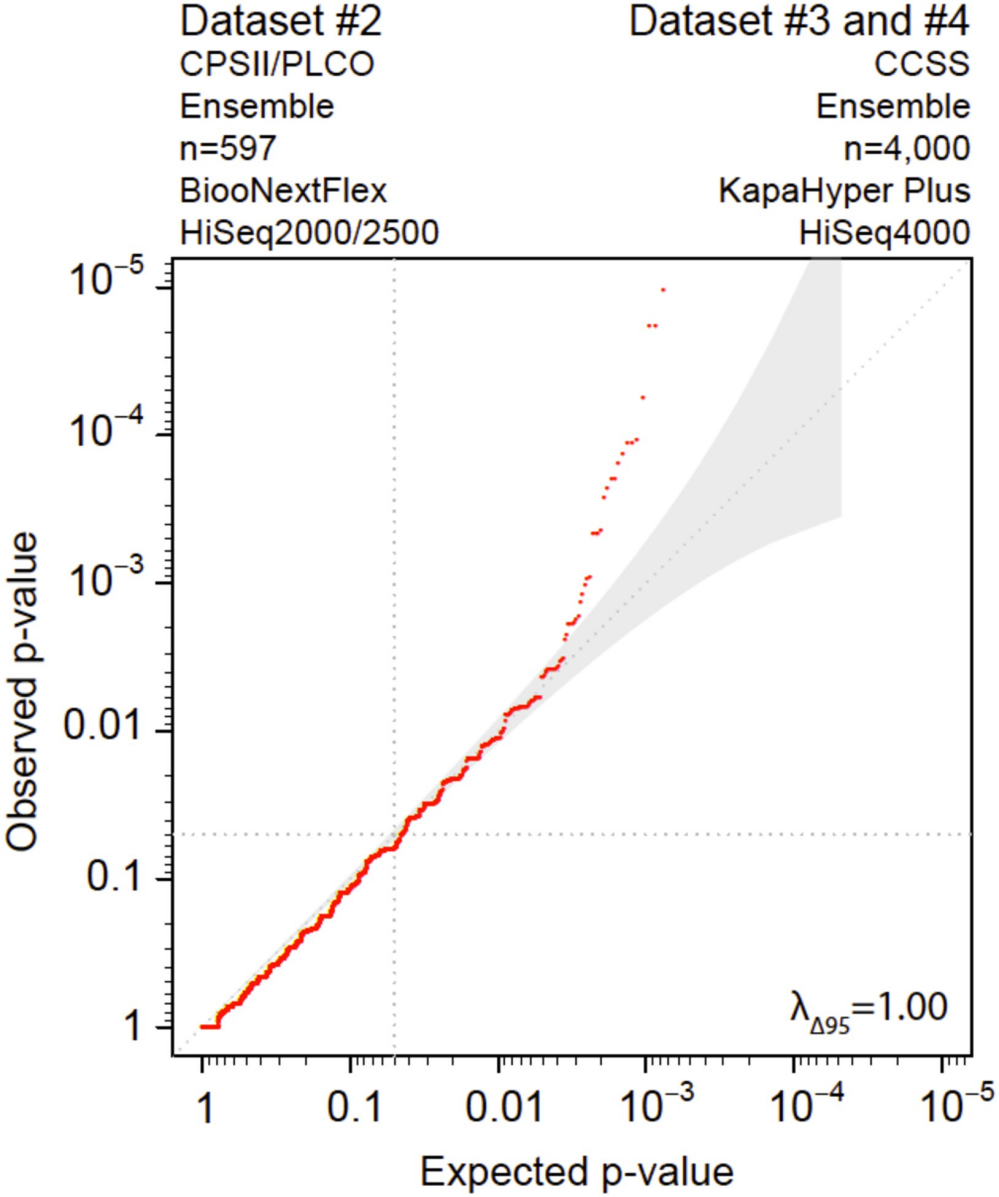
Evaluation of laboratory factors contributing to elevated false-positive findings. Quantile-Quantile plot of two experimental datasets (sub-sampled CCSS data) that used the same capture kit (EZ Exome+UTR PE) and differ in the use of library prep kit (BiooNextFlex vs. KapaHyper Plus) and sequencer (HiSeq 2000/2500 vs. HiSeq 4000). Variants in both cohorts were called using HaplotypeCaller and UnifiedGenotyper and/or Freebayes.

**Fig 3:**
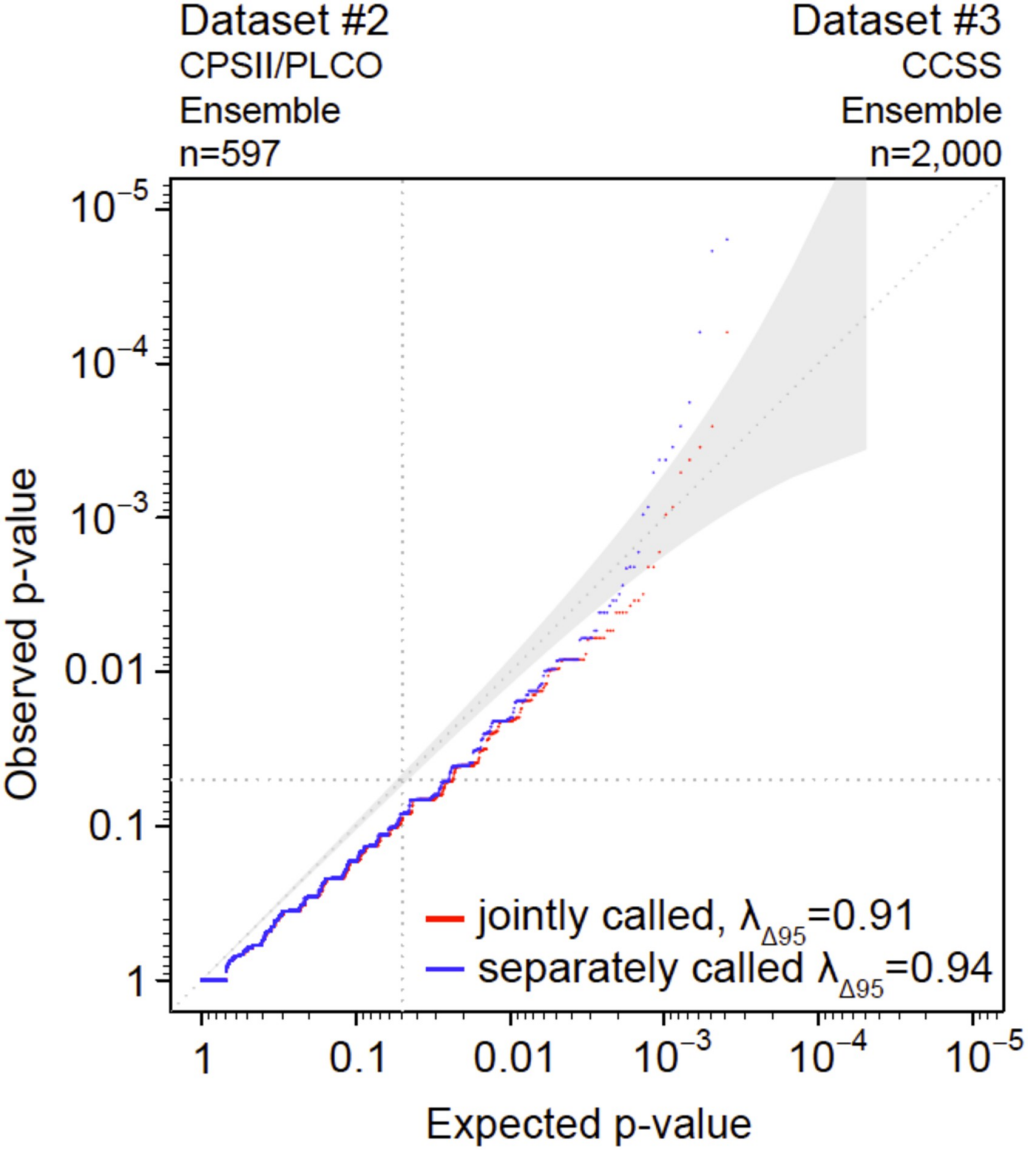
Evaluation of the effects of joint vs separate variant calling on elevated false-positive findings. Quantile-Quantile plot of sub-sampled CCSS data that were called jointly or separately. Red shows the two cohorts variant-called jointly; blue shows the two cohorts variant-called separately.

**Fig 4:**
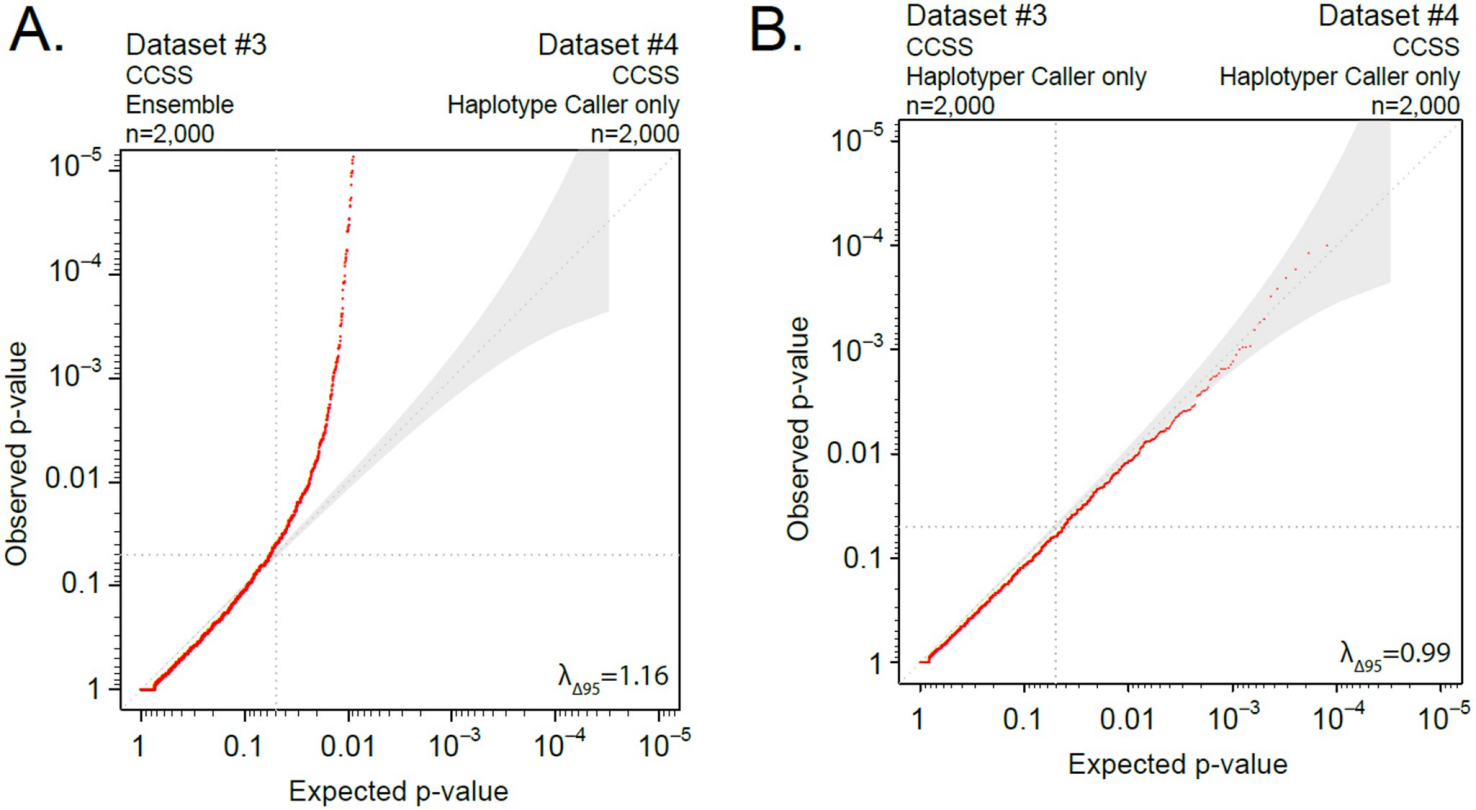
Use of different (A) and same (B) variant-calling pipelines. Quantile-quantile plot of distribution of p-values from synonymous variants in sub-sampled CCSS data (n = 4000) randomly divided (n = 2000 each) and called with (A) different callers (Ensemble vs. HaplotypeCaller) or (B) same caller (HaplotypeCaller) and post-variant filtering. In panel A, we observed inflated p-values deviating from the 95% confidence interval (shading), while in panel B, we observed no deviation from the 95% confidence interval (shading), consistent with minimal or no inflation of p-values.

Table 1 and Figs 1–4 summarize the analyses performed to systematically investigate factors that could contribute to false-positive findings by determining λ_Δ95_, which quantifies the degree of inflation in statistical significance. λ is a metric developed for measuring p-value inflation in genome-wide association studies. Guo *et al*. [6] adapted λ for use in rare-variant association studies to calculate λ_Δ95_ which adjusts for many results with p = 1.00. However, λ_Δ95_ does not fully capture the inflated distribution of test statistics if the observed p-values deviate from expected p-values greater than the median. Thus, visual inspection of the line deviation from the 95% confidence interval (CI, gray area in figures) was also evaluated.

**Table 1:** Summary of data analyses.

	Analysis	Sample Set #1 (n = participants)	Caller for Sample Set #1	Sample Set #2 (n = participants)	Caller for Sample Set #2	Number of Genes Tested	λ_Δ95_
Fig 1	Demonstration of increased false-positive findings with expected-null findings using public controls	Dataset #1: NFE non-TCGA gnomAD (n = 51,377)	HaplotypeCaller	Dataset #2: CPSII/PLCO (n = 597) 97.4% samples have > 95%CEU	HaplotypeCaller	17,482	1.09
Fig 2	Evaluation of laboratory factors contributing to elevated false-positive findings ^1^	Dataset #2: CPSII/PLCO (n = 597)^2^ 97.4% samples have > 95%CEU	Ensemble	Datasets #3 and 4 combined: CCSS (n = 4,000)^3^ 94.9% samples have > 95%CEU	Ensemble	10,461	1.00
Fig 3	Evaluation of the effects of joint vs separate variant calling on elevated false-positive findings	Dataset #2: CPSII/PLCO (n = 597) 97.4% samples have > 95%CEU	Ensemble	Dataset #3: CCSS (n = 2,000) 94.5% samples have > 95%CEU	Ensemble	Joint: 10,244 Separate: 10,224	Joint^4^: 0.91 Separate^5^: 0.94
Fig 4A	Use of different variant-calling pipelines	Dataset #3: CCSS (n = 2,000) 94.5% samples have > 95%CEU	Ensemble	Dataset #4: CCSS (n = 2,000) 95.3% samples have > 95%CEU	HaplotypeCaller	16,281	1.16
Fig 4B	Use of same variant-calling and post-variant filtering	Dataset #3: CCSS (n = 2,000) 94.5% samples have > 95%CEU	HaplotypeCaller	Dataset #4: CCSS (n = 2,000) 95.3% samples have > 95%CEU	HaplotypeCaller	16,327	0.99
S1 Fig	Evaluation of published methods to correct an elevated false positive rate	Dataset #1: NFE non-TCGA gnomAD (n = 51,377)	HaplotypeCaller	Datasets vary: CCSS (n = 4,300, n = 1,000, n = 400) 94.9% samples have > 95% CEU in 4,300 and 1,000 cases and 95.3% samples have > 95.3% CEU in 400 cases	Ensemble	For each method (n = 4,300, n = 1,000, n = 400): TRAPD (14,972, 14,714, 14,710); ProxECAT (3,987, 441, 65); iECAT (12,906, 7,234, 4,898)	For each method, (n = 4,300, n = 1,000, n = 400): TRAPD (1.59, 1.00, 1.35);ProxECAT (2.79, 2.13, 1.90); iECAT (1.20, 0.45, 0.43)

“Ensemble” caller refers to the use of HaplotypeCaller *and* UnifiedGenotyper *and/or* FreeBayes. CPSII: Cancer Prevention Study II (dataset); CCSS: Childhood Cancer Survivor Study (dataset); gnomAD: The Genome Aggregation Database; NFE: Non-Finnish European; PLCO: Prostate, Lung, Colorectal, Ovarian Cancer (dataset); TCGA: The Cancer Genome Atlas. The “Number of Genes Tested” varies since at least five variants per gene is required to be considered.

For each analysis, we evaluated the distribution of rare, synonymous variants from two different sample sets (listed as “Sample Set #1” and “Sample Set #2” which vary depending on the study) collapsed by gene from individuals of non-Finnish European (NFE) ancestry. The caller for the two sample sets also varied, depending on the investigation but was either HaplotypeCaller or “Ensemble,” a combination of three different callers (see S1 File). The number of genes tested varies due to differences in datasets and filtering. We did not perform burden tests on genes with fewer than 3 observed variant carriers. This means that some genes that were testable on larger datasets or with more permissive filtering may no longer be testable with smaller samples or stricter filtering.

We used the exome sequencing component of gnomAD, excluding cases from The Cancer Genome Atlas, (TCGA) (n = 51,377) [3] as a public control dataset. The three exome sequencing datasets from our laboratory were the Prostate, Lung, Colon, Ovary Screening Trial (PLCO) (n = 374) [9], Cancer Prevention Study II (CPSII) (n = 223) [10], and the Childhood Cancer Survivor Study (CCSS) (n = 5,105), [11] all of which are available through dbGaP.

For each figure, the datasets, callers and number of genes in the analysis are listed at the top, which matches the information for each analysis in Table 1. Each figure is a QQ plot, which compares the expected with the observed p-value (on a log scale) for rare, synonymous variants from two different sample sets. Since the evaluated variants are synonymous, we do not expect any deviation from a uniform distribution of p-values under the null hypothesis of no association between phenotypes and rare variants. Thus we expect the slope of the plot to approximate 1. Deviations from the slope = 1 (as measured by visual inspection or λ_Δ95_) suggest systematic noise or error (*e*.*g*., laboratory processes or factors in variant-calling or annotation) with “inflated” p-values. Factors (e.g., use of identical processes for datasets) that reduce inflation restore the compared distributions to a slope that approximates 1.

### Demonstration of increased false-positive findings with expected-null findings using public controls (Table 1, row 2, and Fig 1)

To illustrate the increase in false positive findings using public controls, we compared two ancestry-matched non-disease cohorts using a rare-variant association (“burden”) test of synonymous variants (only) that would be expected to be null. We analyzed variants from 17,482 genes from the 51,377 individuals in the NFE non-TCGA gnomAD dataset and compared them with variants from 597 cancer-free individuals in the CPSII/PLCO cohort. HaplotypeCaller was used for both sample sets, but different post-variant filtering methods were applied. We observed significantly inflated p-values (λ_Δ95_ = 1.09) with a distribution that was highly deviated from the 95% CI in the quantile-quantile (QQ) plot (Fig 1).

### Evaluation of laboratory factors contributing to elevated false-positive findings (Table 1, row 3, and Fig 2)

We next investigated the possible origins of the inflated p-values by focusing on factors that could differ between public controls and an experimental dataset such as laboratory processes (*e*.*g*., capture kit, library prep kit, sequencing platform). To do this, we compared the distribution of rare synonymous variants from 10,461 genes in a dataset from CPSII/PLCO (n = 597) with a dataset from CCSS (n = 4,000) that shared the same capture kit, calling and post-variant filtering but differed in library prep kit and sequencing platform. Although there was some deviation from the 95% CI (41 genes), Fig 2 shows minimal deviation (λ_Δ95_ = 1.00) from the expected null distribution in this comparison.

### Evaluation of the effects of joint vs separate variant calling on elevated false-positive findings (Table 1, row 4, and Fig 3)

We next investigated the possible origins of the inflated p-values by focusing on factors that could differ between public controls and an experimental dataset such as variant-calling differences (*e*.*g*., single *vs*. multiple callers, joint *vs*. separate calling, same *vs*. different callers). To do this, we evaluated the effects of joint *vs*. separate variant-calling on the inflated distribution of test statistics by comparing the distribution of rare synonymous variants from 10,244 genes in a dataset from CPSII/PLCO (n = 597) with a dataset from CCSS (n = 2,000) using the same Ensemble (HaplotypeCaller plus at least one other caller) variant-calling pipeline. Fig 3 shows minimal deviation from the null distribution with variant-calling performed either jointly (λ_Δ95_ = 0.91; both sample sets variant-called together) or separately (λ_Δ95_ = 0.94; each sample set variant-called separately). Taken together, these results suggest that joint *vs*. separate variant-calling does not contribute to the observed inflation.

### Use of different variant-calling pipelines (Table 1, row 5, and Fig 4A)

We next considered the use of different variant-calling pipelines. We randomly separated an experimental dataset derived from CCSS (n = 4,000, samples sequenced at the same time in our laboratory) into two groups (each n = 2,000). Dataset #3, specified in Table 1, row 5, was called using the Ensemble caller, whereas Dataset #4 was called using just HaplotypeCaller. There was a deviation from the null distribution in the QQ plot (λ_Δ95_ = 1.16; Fig 4A) when these two different variant-calling pipelines were used in these datasets.

### Use of same variant-calling and post-variant filtering (Table 1, row 6, and Fig 4B)

To evaluate same variant-calling pipelines, we used Dataset #3 and Dataset #4 (Fig 4A, Table 1, row 6) called using HaplotypeCaller. There was minimal deviation from the null distribution in the QQ plot (λ_Δ95_ = 0.99; Fig 4B) when same variant caller with the same post-variant filters were used on both datasets, illustrating the importance of applying the same variant-calling pipeline and post-variant filtering to compared cohorts.

### Evaluation of published methods to correct an elevated false positive rate (Table 1, row 7, and S1 Fig)

To determine the ability of three published methods (TRAPD [6], ProxECAT [7], and iECAT [8]) to adjust inflated p-values in larger datasets, we analyzed the distribution of rare variants in NFE non-TCGA gnomAD (n = 51,377) with sub-sampled CCSS data of varying sizes (n = 4,300, n = 1,000, n = 400) thus mimicking the methods that were presented in each tool. For the largest dataset (n = 4,300, red lines), we observed highly inflated p-values (λ_Δ95_ = 1.59 [TRAPD]; λ_Δ95_ = 2.79 [ProxECAT]; λ_Δ95_ = 1.20 [iECAT]: Table 1 and S1 Fig). Since the size of the sub-sampled CCSS data examined was larger than in the previously published studies (range n = 393 to 927), [6–8] we decreased the sub-sampled CCSS data from 4,300 to 1,000 and 400. A reduction of the inflated p-values was observed with decreasing sub-sampled dataset size, despite retaining the same set of gnomAD controls (S1 Fig, blue and black lines). This observation suggests that smaller sub-sampled datasets are not powered to detect inflated p-values and that, unfortunately, the currently available methods do not always sufficiently adjust for the increased false-positive findings.

## Discussion

Our analyses of a limited number of datasets show that false-positive results occur if rare-variant association tests are conducted using cases and controls that have different variant-calling and post-variant-calling filtering pipelines. Differences in laboratory components (*e*.*g*., capture kit, library prep kit and/or sequencing platform) and joint *vs*. separate variant-calling did not substantially inflated distribution of test statistics, a finding reported by other groups [12]. Occult population stratification is not a likely explanation for our findings given the very high percentages of European (CEU) ancestry in both case and control cohorts (Table 1). These results, especially if replicated, emphasize the risks of using public controls for association tests in which individual-level data and the computational pipeline are not readily accessible, which prevents the use of the same variant-calling and filtering pipelines.

Possible options to effectively utilize publicly available genomic datasets without introducing substantial biases include: 1) obtaining individual level data from a publicly available dataset and process using the experimental dataset’s variant-calling pipeline through a portal that protects identifying information as per the ethical oversight of the study; 2) access to sufficiently detailed variant-calling and filtering pipeline documentation on publicly available datasets and applying this to the experimental dataset; or, 3) sequencing controls in-house and match the variant-calling pipeline elements with the experimental dataset. However, each of these proposed solutions have limitations, including: 1) lack of adequate consent and/or data-sharing agreements to provide individual-level data from public resources; 2) inadequate computational resources (*e*.*g*., storage and/or processors) needed to process experimental datasets and publicly available resources using the same bioinformatic pipelines; and 3) absence of available in-house controls and/or insufficient resources to sequence and process the resultant data.

Another option is the development and use of a standard variant-calling pipeline by all investigators. However, this poses significant, practical obstacles including the need for continual adjustments to improve accuracy and performance. Moreover, the rapid dissemination of next-generation sequencing technologies has led to many local solutions, making it difficult to develop an academic standard. Until there is a stable solution to compare a dataset to public controls, investigators should carefully evaluate the use of publicly available data for biases and implement strategies and methods to minimize such biases particularly when using a statistical test (*e*.*g*., Fisher’s exact test). At a minimum, public controls should not be the sole dataset in rare-variant association tests.

In summary, public controls are important tools for rare-variant analyses (*e*.*g*., population filtering and variant frequency) but their use for direct statistical tests (*e*.*g*., rare-variant association tests) without the same variant-calling and post-calling variant filtering pipeline is problematic. Importantly, the currently published methods do not adequately adjust for the likely high false-positive findings. A plausible solution exists with the emergence of cloud-based computing, which can make it possible to bring containerized analytical pipelines to the data (rather than the data to the pipeline) and could avert the issues mentioned above. It is suggested that future reports account for this issue and provide this as a limitation in reporting new findings based on studies that cannot practically analyze all data on a single pipeline.

## Materials and methods

(See also S1 File for additional details on selection of datasets, calling and filtering overview, and rare-variant association (burden) testing and assessment.)

### Datasets

Analyses were performed on datasets from previously published large, exome-sequenced cancer cohorts. A dataset of 4,300 long-term cancer survivors was utilized from the Childhood Cancer Survivor Study (CCSS) [11]. Additionally, an in-house control dataset was composed of the combined control sets from the Cancer Prevention Study II (CPSII) [10], and the Prostate, Lung, Colorectal, Ovarian Cancer (PLCO) [9] datasets. To ensure homogenous ancestry, the CPSII [10], CCSS [11] and PLCO [9] datasets were restricted to samples that were estimated to be at least 80% European (CEU) ancestry as determined by industry-standard methods detailed elsewhere [13]. For CCSS, we also restricted samples to those that were not whole-genome-amplified.

Our public control set was composed of publicly available data from the Genome Aggregation Database (gnomAD) [3] v2.1 and including only non-Finnish European (NFE) after excluding data from individuals from The Cancer Genome Atlas (TCGA) (n = 51,377). QQ plots were used to visually demonstrate p-value inflation and the λ_Δ95_ statistic was used for quantitative assessment of this inflation. Details of λ_Δ95_ statistic calculation is in S1 File.

### Variant calling

For datasets called by HaplotypeCaller, the following additional filters were applied (these are the standard hard filters recommended by GATK): QD≥2, FS≤60, MQ≥40, MQRankSum≥-12.5, ReadPosRankSum≥-8, SOR≤3.

For datasets called by Ensemble, the following additional filters were applied: at the genotype level: 1) variants required a GQ > 20 and the alternate allele depth (AD) to be greater than 1, and 2) variant must be called by HaplotypeCaller and either FreeBayes or UnifiedGenotyper. Among heterozygous genotype calls, the total ratio between alternate AD and total depth (DP) must be greater than 0.3. If there were 3 or fewer heterozygous genotype calls, the depth must be greater than 0.2, the observed carrier frequency must be less than 10%, and there must not be any multiallelic heterozygous genotype calls (no individuals with a genotype containing two different alternate alleles).

### Variant filtering and annotation

Variants used in the analyses were 1) classified as synonymous (coding) for at least one gene, 2) not be SNPEFF HIGH or MODERATE for any gene, 3) have an allele frequency less than 0.01 in the population databases (all populations in 1000 Genomes, ESP, and all populations other than NFE in ExAC and gnomAD-exome), 4) within 5bp of the target region, called by HaplotypeCaller and either FreeBayes or UnifiedGenotyper, and 5) must not be a duplicate variant (due to indel alignment issues). At the genotype level, variants were required to have a GQ score greater than 20 and the alternate allele depth to be greater than 1. Among heterozygous genotype calls, the total ratio between an alternate AD and DP must be greater than 0.3, or if there are 3 or fewer heterozygous genotype calls the depth must be greater than 0.2. The observed carrier frequency must be less than 10%. There must not be any multiallelic heterozygous genotype calls. i.e.: no individuals with a genotype containing two different alternate alleles.

### Analyses performed

Five sets of analyses were performed, corresponding to Figs 1–4 plus S1 Fig, as listed in Table 1 and corresponding to section headers in the Results section:

### Demonstration of increased false-positive findings with expected-null findings using public controls

To demonstrate the inflated p-values present in a presumed-null analysis, we compared the Non-Finnish European (NFE) and non-TCGA subset of gnomAD (n = 51,377) with an in-house control dataset (CPSII/PLCO, n = 597; 97.4% of samples have >95% CEU [European] ancestry) using Fisher’s exact test. The following filters were applied: the variant must be 1) called by HaplotypeCaller, 2) within 5 base pairs of the CCSS target region, 3) synonymous and within a coding exon, 4) have an allele frequency less than 0.001 in the population databases (all populations in 1000 Genomes and ESP, and all populations other than NFE in ExAC and gnomAD-exome; as 1000 Genomes and ESP were included as a filtering given a small proportion of the full gnomAD-exome dataset), 5) exist in both the CCSS dataset and the gnomAD dataset, 6) pass the HaplotypeCaller hard filters recommended by the Broad Institute, 7) must not be a duplicate variant (due to indel alignment issues), and 8) must not be on a RepeatMasker SimpleRepeat or a 5-base-pair (or longer) homopolymer run. In addition, 90% of all samples in both CCSS and gnomAD must have coverage depth greater than 10.

### Evaluation of laboratory factors contributing to elevated false-positive findings

To determine whether laboratory factors contributed to p-value inflation, we tested Dataset #2 (CPSII/PLCO control dataset (n = 597)) against Datasets #3 and #4 of the CCSS dataset (n = 4000), again restricting to synonymous coding variants (which presumably would not vary significantly between the two groups). The CPSII/PLCO dataset used the BiooNextFlex library prep kit and was sequenced on a combination of the Illumina HiSeq 2000 and HiSeq 2500 sequencer. The CCSS dataset used the KapaHyper Plus library prep kit and the HiSeq 4000 sequencer.

### Evaluation of the effects of joint vs separate variant calling on elevated false-positive findings

In joint calling, all samples in a dataset are called simultaneously, using information from across all samples to assist in assessing and calling variant loci. Obviously, our datasets and the gnomAD external control dataset were called separately, so we developed a test to determine whether this could be a source of the inflation. A subset of the CCSS dataset (Dataset #3, n = 2,000) and CPSII/PLCO (dataset#2, (n = 597)) were called jointly and separately followed by rare-variant association (burden) tests. The same filter set was used as used in Analysis set 3 (above). Variants used in this analysis must be classified as 1) synonymous (coding) for at least one gene and must not be SnpEff HIGH or MODERATE for any gene, 2) have an allele frequency less than 0.001 in the population databases (all populations in 1000 Genomes, ESP, ExAC, gnomAD exome and gnomAD genome), 3) within 5bp of the target region, 4) called by HaplotypeCaller and either FreeBayes or UnifiedGenotyper, and 5) must not be a duplicate variant (due to indel alignment issues). At the genotype level, variants were required to have a genotype quality (GQ) score greater than 20 and the alternate allele depth to be greater than 1. Among heterozygous genotype calls, the total ratio between alternate allele depth and total depth must be greater than 0.3, or if there are 3 or fewer heterozygous genotype calls the depth must be greater than 0.2. The observed carrier frequency must be less than 10%. There must not be any multiallelic heterozygous genotype calls. ie: no individuals with a genotype containing two different alternate alleles.

### Use of different and same variant-calling pipelines

To determine whether differences in variant-calling methodology could introduce p-value inflation, we split the CCSS dataset into two equally sized subsets (Datasets #3 and #4, n = 2000 each) and ran rare-variant association (burden) tests in which the calling methods differed (Fig 4A: Ensemble and HaplotypeCaller) and in which the calling methods were the same (Fig 4B: HaplotypeCaller only). Variants used in these analyses must be 1) classified as synonymous for at least one gene, 2) must not be SnpEff HIGH or MODERATE for any gene, 3) have an allele frequency less than 0.001 in the population databases (all populations in Thousand Genomes, ESP, ExAC, gnomAD exome and gnomAD genome), 4) within 5bp of the target region and 5), must not be a duplicate variant (due to indel alignment issues).

### Evaluation of published methods to correct an elevated false positive rate

TRAPD, [6] ProxECAT [7] and iECAT [8] were used as per each method’s published reference. For each method, three analyses were performed on case (CCSS) and public control (gnomAD) data: 1) the full 4,300-sample CCSS set (95.1% of samples have >95% CEU [European] ancestry) vs. gnomAD, 2) a random 1000-sample subset of CCSS vs. gnomAD, and 3) a random 400-sample subset of CCSS vs. gnomAD. No genotype-level filtering was performed because there is no way to implement such filters on the gnomAD dataset since we can only access aggregate frequency-level data. Both cases (CCSS) and public controls (gnomAD) were restricted to European ancestry. For gnomAD, specifically the “non-Finnish European” (NFE) without TCGA subset was used. The following filters were applied to both the CCSS and gnomAD datasets for all analysis: 1) variant must be within 5 base pairs of the CCSS target region, 2) must not be a duplicate read, 3) must not be on RepeatMasker, SimpleRepeat or a 5-base-pair (or longer) homopolymer run, and 4) must be called by HaplotypeCaller.

Specific details for each of the three methods are provided in S1 Text in S1 File.

## Supporting information

S1 Fig Evaluation of published methods to correct an elevated false positive rate. Quantile-quantile plot of non-Finnish European non-TCGA (The Cancer Genome Atlas) gnomAD subjects (n = 51,377) versus a sub-sampled CCSS dataset showing greatly inflated p-values, which diminishes with decreasing dataset size. Filtered to include rare variants using methods described in A) TRAPD, B) ProxECAT, C) iECAT.
(TIF)

S1 File Supplemental method. (PDF)
