# Supplementary figures and images for: Inflated expectations: Rare-variant association analysis using public controls
Source: PLoS One. 2023 Jan 25;18(1):e0280951. doi: 10.1371/journal.pone.0280951 (PMC9876209; doi:10.1371/journal.pone.0280951)

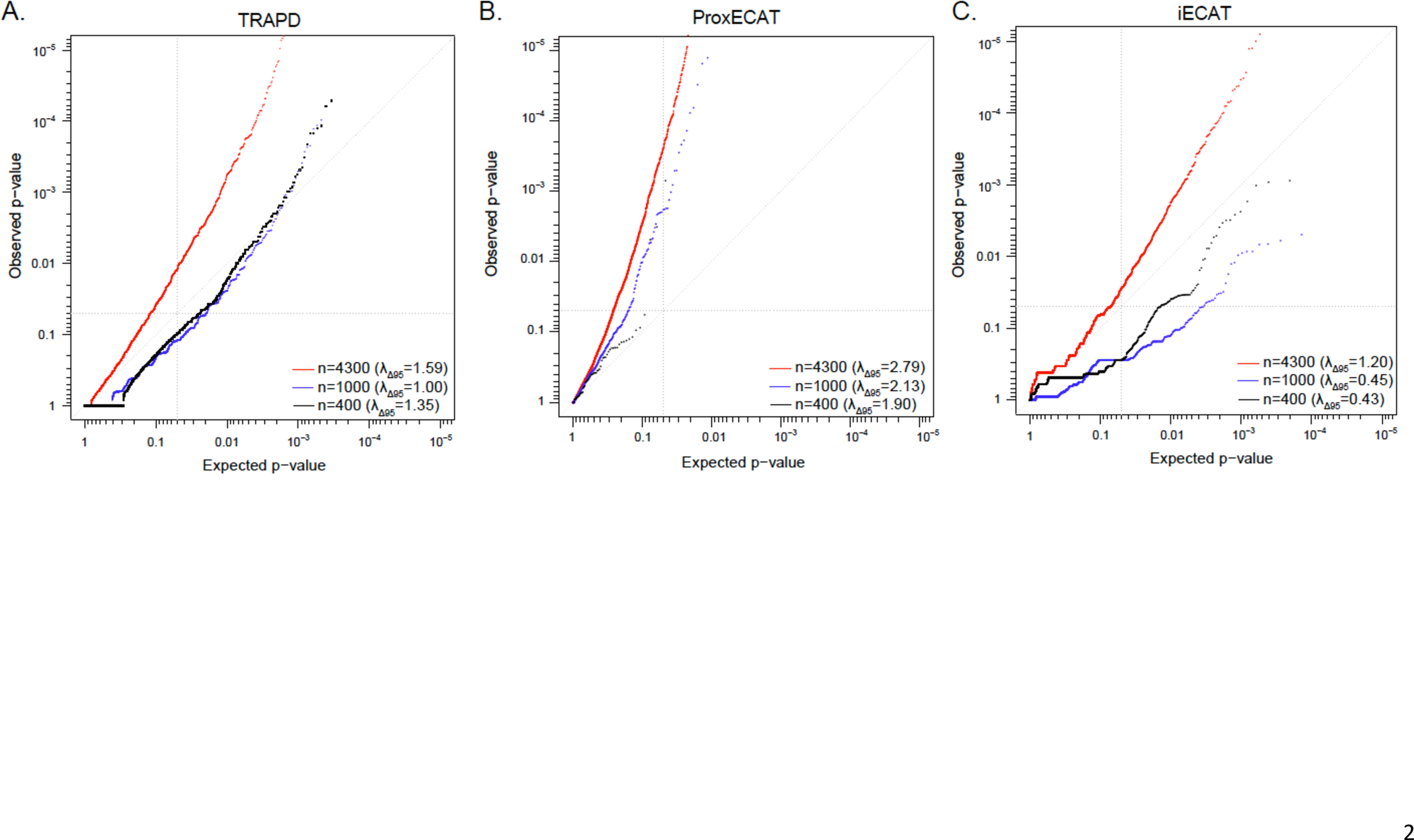

Supplement: S1 Fig — Quantile-quantile plot of non-Finnish European non-TCGA (The Cancer Genome Atlas) gnomAD subjects (n = 51,377) versus a sub-sampled CCSS dataset showing greatly inflated p-values, which diminishes with decreasing dataset size. Filtered to include rare variants using methods described in A) TRAPD, B) ProxECAT, C) iECAT. (TIF) [file pone.0280951.s001.tif]
