# Supplementary material for: Inflated expectations: Rare-variant association analysis using public controls
Source: PLoS One. 2023 Jan 25;18(1):e0280951. doi: 10.1371/journal.pone.0280951 (PMC9876209; doi:10.1371/journal.pone.0280951)
Supplement: S1 File — (PDF) [file pone.0280951.s002.pdf]

Supplemental Information

S1 Text. Supplemental method

Selection of datasets

Seven distinct sample sets were used:

To summarize, the three base datasets were:

- Dataset #1: gnomAD NFE non-TCGA external control dataset (n=51,377)
- Dataset #2: CPSII + PLCO combined control dataset, CEU>0.8 (n=597)
- Datasets #3 and 4: CCSS long-term cancer survivor dataset, CEU>0.8, non-whole-genome-amplified (n=4,300)

Additionally, four random subsets were drawn from the CCSS dataset:

- For TRAPD, ProxECAT, and iECAT analyses:
  - Subset 1: n=1,000
  - Subset 2: n=400
- Datasets #3 and #4: Two mutually exclusive subsets consisting of a random partition of subset 3 into two equal halves (n=2000 each).

Calling and filtering overview

The exact filter set used varied considerably depending on the analysis. In particular, many of the filters that we have found to be effective in eliminating erroneous variant calls rely on information that is not available in the external control datasets, and thus these filters could not be applied in those analyses. In order to maintain comparability, the same filters were applied to both cases and controls in each analysis unless specifically noted otherwise.

In analyses composed entirely of internally generated data, all samples in a given analysis were jointly called together as a single dataset (unless specifically noted otherwise as in analysis 4 which specifically tests for a potential joint-calling effect).

Some variant filters were applied uniformly to all analysis: 1) duplicate variants were removed, 2) variants were required to be within 5 bp of the exome target region, and 3) variants must have an alternate allele called that passes the genotype filter (if applicable). Note that the "target region" refers to the target region for our own sequencing datasets, the gnomAD dataset is composed of a heterogenous combination of at least ten different capture kits (https://gnomad.broadinstitute.org/news/2018-10-gnomad-v2-1/).

In applicable analyses, the "Ensemble" caller refers to a variant callset derived from the combination of variant calls from three different callers: HaplotypeCaller (HaplotypeCaller using GATK3.1-1q, CombineGVCFs using GATK3.1-1, and GenotypeGVCFs using GATK3.3-0) and UnifiedGenotyper (GATK 3.1-1) and FreeBayes (freeBayes v0.9.14-24-gc292036 and GATK selectvariants 3.1-1). Final variant calls were generated using this "ensemble" of variant calls, genotype-level filters were also applied, requiring that the genotype quality (GQ) be greater than or equal to 20 and that the alt allele depth be greater than or equal to 2.

Other filtering statistics that were commonly applied included the "hetAB", which is defined as the ratio between the observed total depth on the alt allele and the total depth across all alleles,

restricted to samples where the final genotype call is heterozygous. In our analysis, if information is available, hetAB cut-off of 0.3 was used. The hetAB is often a useful proxy for false variant calls, as sequencing, library prep, mapping, or calling artifacts often do not produce alt allele depths that are balanced with the reference allele depth.

For HaplotypeCaller only, GATK "hard filters" were used (https://gatk.broadinstitute.org/hc/en-us/articles/360035890471-Hard-filtering-germline-short-variants). This consisted of the following filters for single nucleotide variants: QD≥2, FS≤60, MQ≥40, MQRankSum≥-12.5, ReadPosRankSum≥-8, and SOR≤3, and the following filters for indel variants: QD≥2, FS≤200, MQRankSum≥-12.5, ReadPosRankSum≥-20, and SOR≤10.

In analyses comparing to gnomAD, a depth filter was applied to cases and gnomAD controls, requiring that 90% of all samples in ***both*** datasets have total coverage depth of at least 10. For the gnomAD set this was determined using the publicly available coverage depth summary tables provided by gnomAD. For the internal datasets this was determined by calculating the coverage depth for each sample and each base-pair locus using the QoRTs quality control and data processing tool[1].

Rare-variant association (burden) testing and assessment
The $\lambda_{\Delta 95}$ statistic, first introduced in Guo et. al[2], is a metric intended to measure the degree of inflation at the tail end of the QQ plot. It is analogous to the lambda inflation factor commonly used to describe the degree of inflation due to population stratification. The traditional lambda compares the observed p-value to the expected p-value at the median, however the inflation we observe here is mostly concentrated on the tail end of the distribution, so the traditional lambda is insufficient. The $\lambda_{\Delta 95}$ statistic is analogous but rather than comparing the median point, it compares the 95th percentile. The formula is:

$$\lambda_{\Delta 95} = \frac{(-\log(P_{obs95})) - (-\log(P_{obs0}))}{\left(-\log\left(P_{exp95}\right)\right) - \left(-\log\left(P_{exp0}\right)\right)}$$

Where P_{obs95} and P_{exp95} are the observed and expected p-values at the 95th percentile point. P_{exp0} and P_{obs0} are the expected and observed p-values for the points where the observed p value is equal to 1.
Burden tests, QQ-plots, $\lambda_{\Delta 95}$ calculation, and similar were all performed in R 3.6.0.
In all analyses, burden tests were restricted to genes with at least 5 observed alt genotype calls and in which the number of observed alt genotype calls was less than 25% of the overall sample set.

Dataset analyses
TRAPD: The following is a complete list of the base filters used for both datasets: 1) 90% of all samples in both CCSS and gnomAD must have coverage depth greater than 10, 2) variant must by synonymous and within a coding exon, 3) an allele frequency less than 0.001 in 1000 Genomes and ESP, and all populations other than NFE in ExAC and gnomAD-exome, and 4) exist in both the CCSS dataset and the gnomAD dataset

As per the TRAPD methodology described in Guo et al[2], we tested various combinations of the QD threshold in order to find the optimal pair of thresholds that minimizes observed p-value inflation. We tested 21 different QD thresholds: QD>1, 2, 3, 4, 6, 8, 10, 12, 14, 16, 18, 20, 22, 24, 26, 28, 30, 32, 34, 36, and 37. Thus 441 (21 x 21) different analyses were run, with each

possible pairing of QD thresholds for the cases and for the controls, respectively. From among these 441 analyses the optimal combination of QD thresholds was determined by finding the combination that yielded the lowest $\lambda_{\Delta 95}$ value. The optimal thresholds were found to be QD>14, QD>16, and QD>14 for the CCSS-full, CCSS-1000, and CCSS-400 datasets respectively. For gnomAD, the optimal threshold was found to be QD>6 in all three comparisons.

In a normal TRAPD-based analysis, these optimized thresholds would then be applied to an equivalent analysis of non-synonymous or deleterious variants, with the idea being that the same reduction in inflation observed in the synonymous set would apply and any significant variants would thus be true positives. Since using non-synonymous variants would increase the likelihood of true positive differences appearing, we instead simply show the optimal comparison itself in the synonymous set.

ProxECAT: The following is a complete list of the base filters used specific for this analysis for both datasets: variant must have an allele frequency less than 0.001 in the all populations in 1000 Genomes and ESP, and all populations other than NFE in ExAC and gnomAD-exome. Loss-of-function variants were selected based on Variant Effect Predictor (VEP)[3] annotation. Splice donor/acceptor, stop-gained, and synonymous variants were also selected based on VEP annotations. Synonymous and lost-of-function (functional) variants was run on the ProxECAT R script provided by the authors, with a minimum count of 2 with the weighted function option.

iECAT: CPSII and PLCO datasets were used as internal controls. The following is a complete list of the base filters used for both datasets: 1) variant must have an allele frequency less than 0.001 in gnomAD non-TCGA NFE+internal control+CCSS dataset, 2) exist in both the CCSS dataset and the gnomAD dataset, and 3) be “HIGH” impact variants by snpEff.

Supplemental Information References

1. Hartley, S.W., and Mullikin, J.C. (2015). QoRTs: a comprehensive toolset for quality control and data processing of RNA-Seq experiments. BMC Bioinformatics *16*, 224. 10.1186/s12859-015-0670-5.
2. Guo, M.H., Plummer, L., Chan, Y.M., Hirschhorn, J.N., and Lippincott, M.F. (2018). Burden Testing of Rare Variants Identified through Exome Sequencing via Publicly Available Control Data. Am J Hum Genet *103*, 522-534. 10.1016/j.ajhg.2018.08.016.
3. McLaren, W., Gil, L., Hunt, S.E., Riat, H.S., Ritchie, G.R., Thormann, A., Flicek, P., and Cunningham, F. (2016). The Ensembl Variant Effect Predictor. Genome Biol *17*, 122. 10.1186/s13059-016-0974-4.
